# On the Spatiotemporal Nature of Vision, as Revealed by Covered Bridges and Puddles: A Dispatch from Vermont

**DOI:** 10.1177/20416695211062625

**Published:** 2021-12-23

**Authors:** Gideon Paul Caplovitz

**Affiliations:** Department of Psychology, University of Nevada Reno

**Keywords:** perceptual organization, scene perception, spatiotemporal factors, grouping

## Abstract

Retinal painting, anorthoscopic perception and amodal completion are terms to describe visual phenomena that highlight the spatiotemporal integrative mechanisms that underlie primate vision. Although commonly studied using simplified lab-friendly stimuli presented on a computer screen, this is a report of observations made in a novel real-world context that highlight the rich contributions the mechanisms underlying these phenomena make to naturalistic vision.

Retinal painting, anorthoscopic perception and amodal completion are terms to describe visual phenomena that highlight the spatiotemporal integrative mechanisms that underlie primate vision. Vision scientists have spent over a century studying the mechanisms underlying these phenomena through the use of behavioural (Fendrich & Mack, 1981; Fendrich et al., 2005; Gerbino, 2020; Helmholtz & Southall, 1867/1962; Lorenceau and Shiffrar, 1992; Michotte & Burke, 1951/1962; Schweitzer & Rolfs, 2020; Stanley & Molloy, 1975; Zöllner, 1862;Tyler, 2019) and more recently, neuropsychological (McCarthy et al., 2015; Orlov & Zohary, 2018; Thielen et al., 2019; Yin et al., 2002) experiments in which simplified stimuli were presented on computer monitors. Just as the value of carefully controlled experiments is undeniable, so too is the ennui of studying the boundlessly rich experiences vision offers us within the constrained confines of the testing room. Thus it was with distinct pleasure that I had the opportunity to experience these perceptual phenomena in all their full glory while on a springtime walk across a covered bridge and down a dirt road in Vermont, U.S.A.

Shown in Figure 1 is the Kingsley Covered Bridge in North Clarendon, Vermont. The Kingsley Covered Bridge is on the U.S. National Register of Historic Places, and is a single-span lattice truss structure, built by Timothy K. Horton in the mid 1800s. As can be seen in the photographs, the walls of the bridge contain no windows. From the interior of the bridge one might assume that these windowless walls would obscure views of the scenic Mill River that flows under the bridge. Close inspection of the walls, however, reveals that they were constructed using vertical boards that do not fit perfectly together, thus leaving small vertical gaps between each pair of boards. When viewed while standing in the interior of the bridge these gaps are too narrow to see any significant features of the landscape beyond (Right Photograph). I noticed however that the experience is quite different when I began walking across the bridge! As I walked, the gaps swept across my retinas and the wall itself became transparent, transforming into a window revealing holistic views of the river and tree lined valley beyond. I believe there are several factors underlying this striking phenomenon: primarily visual persistence leading to retinal painting, but also a bit of amodal completion across multiple gaps, and perhaps even a small contribution of anorthoscopic spatiotemporal integration within each gap.

**Figure 1.**
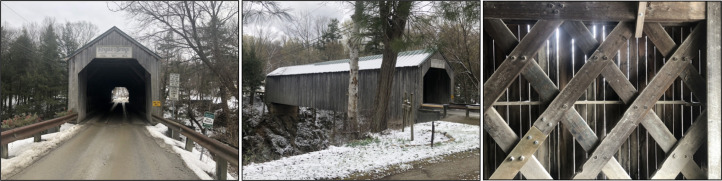
The Kingsley Covered Bridge left and middle images show the historic lattice-truss structure. Right image shows the vertical gaps as seen from the interior of the bridge. Note: While standing still, by default the wall serves as the depth of fixation rendering any potentially visible form-features of the outdoor scene out of focus and nearly invisible. With great effort, I could shift to a distant depth of fixation and experience seemingly random bits and pieces of colour and texture within the gaps.

At slow walking speeds, this window has a darkened transparency and takes on a flickering characteristic not unlike an old movie played slowly, as the persistent high contrast image provided by each gap overlaps the intervening boards. At running speeds or when looking out a car window, which also provides a more stable viewing platform, the flicker is much faster, less intrusive and the image appears brighter, remarkably uniform and stable. This observation is consistent with the Talbot-Plateau Law which asserts the perceived intensity of a flashing light will be determined by the ratio of the on and off periods (Arnold & Winsor, 1934; Plateau, 1830; Talbot, 1834) and would be expected from retinal painting (Scherzer & Ekroll, 2015). That said, at slow walking speeds the persistent images afforded through the gaps were insufficient to fully superimpose with the intervening boards. Despite the corresponding sense of flicker, I could still experience a holistic view of the scene beyond thus highlighting the role of amodal completion in piecing together form information across the gaps. Finally, although I did not have the luxury of an eyetracker to verify the position of my eyes, I find it difficult to believe that my point of gaze was consistent relative to my physical trajectory. As such, I believe it unlikely the uniform and stable image afforded by the widow can be accounted for by strictly orthoscopic mechanisms and thus conclude that the spatiotemporal integration mechanisms that underly anorthoscopic perception (McCarthy et al., 2017), operating locally within the regions of individual gaps, likely play a role as well.

For reasons that I suspect have to do as much with my own incompetence as well as depth of focus, exposure duration and discrete sampling, my attempts to video the effect have proved very ineffective. Demonstration Video 1 provides glimpses of the scene down river from the bridge; although the video completely fails to capture the experience of viewing in person. Figure 2 illustrates my attempt to recreate somewhat more true-to-life examples of the effect in a still image. Given the effectiveness of the window at speeds readily attained by walking or on horseback, I am left to wonder if these small vertical gaps were intentional features of Mr. Horton's 1800′s-era design.

**Figure 2.**
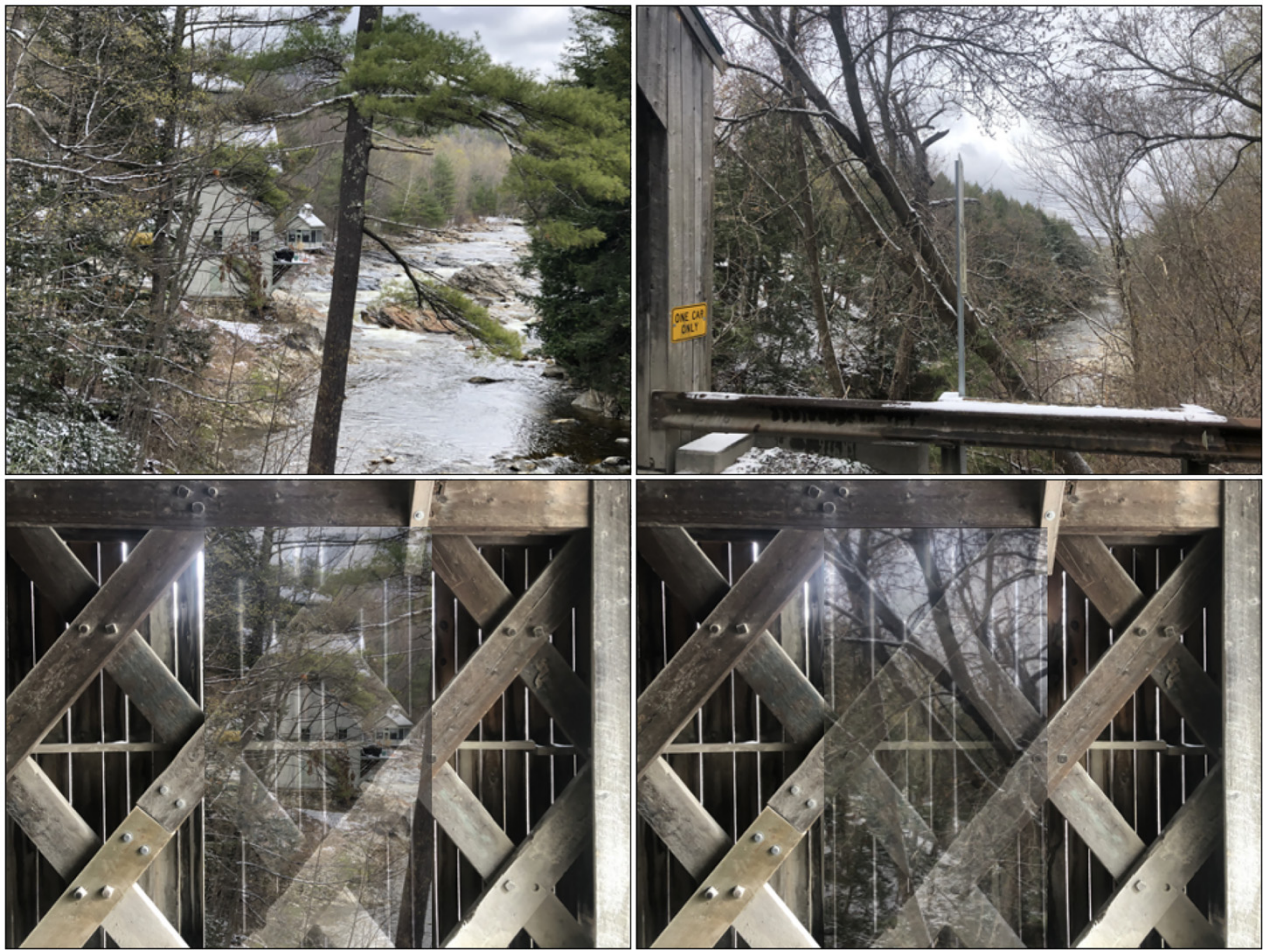
A poor reconstruction of the retinally-painted “window”. The scenes looking up-river (left) and down-river (right) from the bridge become visible from the interior (bottom) when travelling over the bridge. While distinct in the nature of the stimulus, a qualitatively similar experience to the window can be observed by viewing a scene through the rotating blades of a fan.

Winter in Vermont is cold and there is a certain joy that comes with the first signs of warming weather, melting snow and the transition to what locals call “mud season”. Dirt roads covered in ice and snow for months turn into rutted, muddy affairs dotted with puddles. As I continued my walk beyond the covered bridge, I found myself on one such puddle-filled dirt road. When standing still, within the puddles small glimpses can be seen of the reflections of the overhanging branches of the trees that line the road (Figure 3).

**Figure 3.**
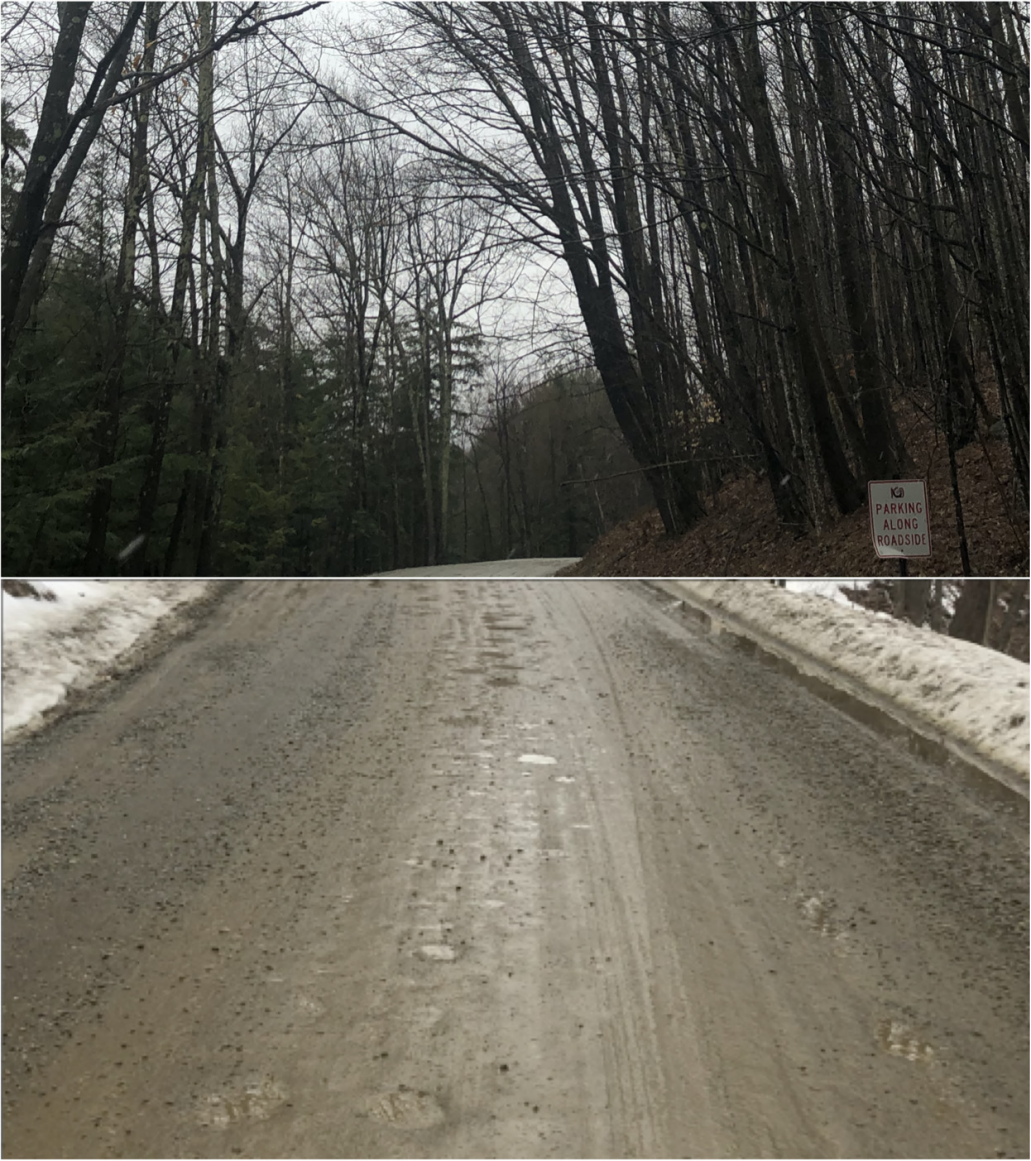
A country road. Top: A tree-lined dirt road in north clarendon, VT. Bottom: In early Spring the winter snows begin to melt and the road becomes covered in small puddles. In a still image like this, one can catch only glimpses of the reflected tree-canopy in the puddles.

Taken as a single snapshot moment in time, these glimpses provide very little sense of canopy above. This is likely because the piecemeal features reflected in each puddle comprise distinct complex textures with little between-puddle collinearity (Gove et al., 1995) or 3D conformation (Tse, 1998) to support amodal completion. However, as I walked along, the reflections revealed more and more of the canopy within each puddle, potentially at times in an anorthoscopic fashion. The dynamic nature of these reflections provide common-fate signals that facilitate contour segmentation and unification and afforded the necessary information to allow amodal completion to ‘kick in’, thus revealing a robust experience of the overhanging branches and sky beyond! For brief moments, I would even get the sense that the road was full of holes like swiss cheese, and I was looking down at a bizarre upside down world rather than a reflect image of the canopy above. My attempts to capture the effect on video were somewhat more fruitful than on the bridge but still do not do justice to the richness of the experience. The brief video clips shown in Demonstration Video 2 and Demonstration Video 3 give a reasonable sense of the percept. I find it interesting to pause the video at various points to contrast how difficult it is to experience the global percept in the absence of motion. Such differences are even more compelling in person and highlight the interaction and contributions of motion to amodal completion. At times when standing still and I suspect with changes in focal distance, the puddles could lose their reflective properties and take on a transparency that reveals their muddy depths, only to have the reflected canopy return when commencing to walk again.

In conclusion, I appreciate the light-hearted nature of visual illusions and the simple demonstrations of perceptual phenomena highlighted in our textbooks, deployed on our vision-laboratory testing computers and sent to us via email by friends and relatives. That said, there is something special about the genuine feelings of happiness when the intricacies of visual perception are revealed to us through real-world experiences like those described here, particularly coming amidst these times of pandemic tragedy. I sincerely hope the reader will enjoy this account of the spatiotemporal integrative mechanisms that underly our visual experiences as much as I have.

## Supplementary Material

Supplementary material

Supplementary material

Supplementary material
